# Socioeconomic, cultural and demographic maternal factors associated with dietary patterns of infants

**DOI:** 10.1016/j.rpped.2015.03.006

**Published:** 2015

**Authors:** Andréa Marques Sotero, Poliana Coelho Cabral, Giselia Alves Pontes da Silva

**Affiliations:** a Universidade de Pernambuco (UPE), Petrolina, PE, Brazil; b Departamento de Nutrição, Universidade Federal de Pernambuco (UFPE), Recife, PE, Brazil; c Pediatria, Universidade Federal de Pernambuco (UFPE), Recife, PE, Brazil

**Keywords:** Food habits, Food consumption, Infant

## Abstract

**Objective:**

To analyze dietary patterns of infants and its association with maternal socioeconomic, cultural, and demographic variables.

**Methods:**

A cross-sectional study was conducted with two groups of mothers of children up to 24 months (*n*=202) living in the city of Maceió, Alagoas, Northeast Brazil. The case group consisted of mothers enrolled in a Family Health Unit. The comparison group consisted of mothers who took their children to two private pediatric offices of the city. Dietary intake was assessed using a qualitative and validated food frequency questionnaire (FFQ). The evaluation of the FFQ was performed by a method in which the overall rate of consumption frequency is converted into a score.

**Results:**

Children of higher income families and mothers with better education level (control group) showed the highest median of consumption scores for fruits and vegetables (*p*<0.01) and meat, offal, and eggs (*p*<0.01), when compared with children of the case group. On the other hand, the median of consumption scores of manufactured goods was higher among children in the case group (*p*<0.01).

**Conclusions:**

Maternal socioeconomic status influenced the quality of food offered to the infant. In the case group, children up to 24 months already consumed industrial products instead of healthy foods on their menu.

## Introduction

Adequate nutrition in the first 2 years of life is essential, as this is a period characterized by rapid growth, development and formation of eating habits that may remain throughout life.^1^^,^ 2

Exclusive breastfeeding up to 6 months and, after this age, breastfeeding up to 2 years or more, combined with the opportune introduction of balanced complementary foods (CF), are emphasized by the World Health Organization as important public health measures, with an effective impact on the decreased risk for development of chronic non-communicable diseases (NCDs) such as obesity, hypertension and diabetes mellitus.^1^^,^ 3 However, studies performed in the last 30 years show that the frequency of NCDs has increased in all Brazilian regions and social classes, especially childhood obesity, with prevalence rates ranging from 5 to 18%.^4^

Feeding practices, in addition to being determinants of health status in children, are strongly associated with the purchasing power of families, as they directly influence the availability, quantity and quality of consumed food.^5^ In recent years, there have been changes in the eating habits of the population, especially regarding the substitution of homemade and natural foods by processed foods, considered superfluous, with high energy density and low nutritional quality.^5^^,^ 6 The advertising market, globalization, the rapid pace of life in big cities and women's work outside of the household have also contributed to these changes.^7^

The identification of dietary patterns of infants is an important study object in nutritional epidemiology, aimed to understand one of the factors responsible for health in childhood.^8^ On the other hand, there is need for improvement in the means of assessing dietary patterns through the use of new methodologies.^9^

Thus, the use of the food consumption frequency method, analyzed through scores, may be a useful tool in the assessment of food quality offered to the infant. Therefore, this study aims to analyze the pattern of food intake of infants and its association with maternal socioeconomic, cultural and demographic variables.

## Method

A cross-sectional study was carried out, involving two groups of mothers of children aged up to 24 months living in the city of Maceió, state of Alagoas, Brazil. The case group consisted of mothers enrolled at the Carla Nogueira Family Health Unit (FHU), located in a low-income neighborhood belonging to the VI Health District. The comparison group consisted of mothers who took their children to two private pediatric offices of the city.

Inclusion criteria were: mothers of children aged up to 24 months and who had a working TV set at home, as television has become the cultural factor that more often disseminates messages about unhealthy foods.^1^The exclusion criteria were: adolescent mothers and, also for the comparison group, mothers who had the doctor's consultation fee paid by another person outside the family.

The convenience sample consisted of 202 mothers of infants, attending study sites and who, in addition to meeting the inclusion criteria, signed the informed consent form between February and April 2012. Of the 220 recruited mothers, 202 mothers participated in the study, with a sample loss of 8.1%. The study was submitted to the Institutional Review Board of Universidade Federal de Pernambuco and it was approved on January 23, 2012, under protocol number 0437.0.172.000-11.

The sample size was estimated using the EPI-INFO software, version 3.5. For the sample calculation, it was considered that 49.0% of the mothers of low socioeconomic status introduce superfluous food at an early age, based on the study by Spinelli, Souza and Souza,^10^ and 28.0% of the mothers, due to better socioeconomic status, do not introduce them, assuming a relative risk of 2.5 of not introducing superfluous food at an early age for a significance level of 95% (1-α) and a power equal to 80% (1-β), with a ratio between the case/comparison group of 1:1. The estimated value of the sample was 92 mother-infant binomials in each group, adding 10% by group to compensate for losses, resulting in a sample of 100 binomials in each group. The final sample comprised a total of 200 mother-infants.

Data collection was performed in the first half of 2012 by two trained nutritionists, who interviewed the biological mothers of children at the FHU and the private offices. The following data were collected: socioeconomic (family income in minimum wages, at the time R$622.00, later distributed into quartiles), cultural (behavior regarding television), demographic (maternal age, occupation, number of live births, mother living with partner, head of the household, maternal and head of the household educational level) and child health (breastfeeding, complementary feeding and prenatal support). For this study, the "breastfeeding" variable was based on the definition adopted by the WHO,^11^ when the child receives breast milk straight from the breast or expressed, regardless of the child receiving other types of foods or not.

Aiming to standardize form completion, we prepared a manual with guidelines for the interviewers and variable codification. The tools were pre-tested with mothers who attended the FHU 30 days before data collection started.

Food intake, the outcome variable, was assessed using a qualitative food frequency questionnaire (FFQ) validated by Colucci et al.,^12^ which was adapted for the age group of children up to 24 months in order to better match the proposed objectives. The FFQ assessment was performed using the method proposed by Fornés et al.,^13^in which the overall rate of consumption frequency is converted into a score.

The scores were set as follows:

1. Foods were recorded through the FFQ, consisting of 63 food items and classified into four consumption categories: rare/never, daily, weekly, or monthly.
2. For the consumption frequency of each food to be considered as monthly consumption, a weight was assigned to each category of consumption frequency.
3. The daily consumption frequency was established as maximum weight value (weight=1). The other weights were obtained according to the following equation: weight=(1/30)×(*a*), where *a*correspond to the number of days consumption occurred during the month. When *a* referred to the weekly consumption, the number was converted to the monthly consumption frequency, multiplying it by 4, considering that the month has 4 weeks. For example, for a food item eaten 4 times in a week, the monthly consumption frequency was 16 times a month. Thus, the weight for the frequency was: weight=(1/30)×(16)=0.533.
4. After calculating the weight of the consumption frequency of each item, the analyzed foods were inserted into six food groups established by the Brazilian Food Guide for children under 2 years^13^: group I consisted of foods that were carbohydrate sources (breads and cereals), which comprise the pyramid base; group II consisted of foods that were sources of vitamins, minerals and fiber (fruits and vegetables); group III consisted of foods that were sources of animal protein and legumes (meat, viscera, eggs and beans); group IV consisted of foods that were sources of calcium (milk and dairy products); group V consisted of foods at the top of the pyramid (sugars, sweets, fats and oils), and group VI consisted of processed foods considered to be superfluous, which, according to the Brazilian Food Guide for children under 2 years, should not be offered to children at this age range, as they have excessive amounts of lipids and/or sugars or contain undesirable substances for consumption by this age group, such as dyes and chemical preservatives (beef broth, industrialized soup, instant noodles, artificial juice, soda, processed sweets, gelatin, sandwich cookies, snacks, buttery popcorn, chocolate milk, candy, sugary breakfast cereals, chocolates, processed baby food, sausages, hot dogs, hamburgers and salami).

The consumption frequency scores were calculated by the sum of the consumption frequency values for foods corresponding to each group. The score I was represented by the sum of consumption values of foods that comprised group I, score II by the sum of consumption values of foods that comprised group II, and so on. However, considering that the food groups established for this study consisted of different numbers of food, the mean score of each group was considered to characterize the food consumption pattern.

The database was entered into the Epi Info software, version 6.04 (CDC/WHO, Atlanta, GA, USA), with double entry, and subsequent use of the validate module to check any typos. For statistical analysis, we used the SPSS version 12.0 (SPSS Inc., Chicago, IL, USA). The chi-square test was used to compare the frequencies between case and comparison groups. Because they are variables in ordinal scale, the frequency scores of food intake were described as medians and interquartile range (IQ). The association between food intake and explanatory variables was assessed by Mann-Whitney *U* test (two medians) and Kruskal Wallis (over two medians), using the Mann-Whitney *U* test subsequently. A *p* value <0.05 was used in the validation of the assessed associations.

## Results

A total of 202 mothers were interviewed: 51% (*n*=103) in the case group, who had their children treated at the Carla Nogueira FHU, and 49% (*n*=99) in the comparison group, who had their children treated at two private offices. Table 1 shows the socioeconomic and demographic characteristics of the study groups.

**Table 1:** Socioeconomic and demographic variables, according to the groups, Maceio-AL, 2012.

Variables	Case			Comparison			Total		*p* a
	*n*	%		*n*	%		*n*	%	
*Maternal age (years)*									
≤28	81	78.6		26	26.3		107	53.0	<0.001
>28	22	21.4		73	73.7		95	47.0	

*Maternal occupation*									
Does not work/homemaker	48	46.6		10	10.3		58	28.7	<0.001
Unemployed	38	36.9		15	14.4		53	26.2	
Employed	17	16.5		74	75.3		91	45.0	

*Number of live births*									
1	32	31.1		58	58.6		90	44.6	<0.001
2 and 3	53	51.5		40	40.4		93	46.0	
>3	18	17.5		1	1.0		19	9.4	

*Mother lives with partner*									
Yes	86	83.5		90	90.9		176	87.1	0.08
No	17	16.5		9	9.1		26	12.9	

*Head of household*									
Mother	10	9.8		11	11.1		21	10.4	0.77
Father	85	82.4		80	80.8		165	81.7	
Grandmother	5	4.9		3	3.0		8	4.0	
Other	3	2.9		5	5.1		8	4.0	

*Maternal schooling (years)*									
<4	5	4.9		0	0.0		5	2.5	<0.001
4–8	96	93.2		16	16.2		112	55.4	
>8	2	1.9		83	83.8		85	42.1	

*Head of household schooling (years)*									
<4	17	16.5		0	0.0		17	8.4	<0.001
4–8	83	80.6		23	23.2		106	52.5	
>8	3	2.9		76	76.8		79	39.1	

*Family per capita income (quartiles of minimum wages)*									
≤0.20	58	56.3		0	0.0		58	28.7	<0.001
0.20–0.55	42	40.8		3	3.0		45	22.3	
0.55–2.71	3	2.9		47	47.5		50	24.8	
≥2.71	0	0.0		49	50.5		49	24.3	

*Child caregiver*									
Mother	99	96.1		62	62.6		161	79.7	<0.001
Maid/nanny	0	0.0		27	27.3		27	13.4	
Others	4	3.9		10	10.1		14	6.9	

a Statistical significance obtained by the Chi-square test.

The mothers in the case group had lower educational levels and family income, were younger when they became mothers and frequently had more than one child when compared to mothers from the comparison group, and these differences were statistically significant (*p*<0.001).

Table 2 shows no difference between the groups regarding the duration of breastfeeding, with approximately 70.0% of children being breastfed for ≤6 months. On the other hand, although the comparison group mothers received less advice on breastfeeding during prenatal care (44.4%×63.0%; *p*<0.01), after birth these mothers were the ones who had more help with the breastfeeding process (*p*<0.001) and higher volume of advice on breastfeeding (*p*=0.04) and the introduction of new foods (*p*<0.001), when compared to the case group mothers.

**Table 2:** Behavioral and nutritional advice variables related to the children's health, according to the case group and comparison, Maceio, AL, 2012.

Variables	Case			Comparison			Total		*p* a
	*n*	%		*n*	%		*n*	%	
*Age of child (months)*									
≤13	56	54.4		52	52.5		108	53.5	0.452
>13	47	45.6		47	47.5		94	46.5	

*Breastfeeding duration*									
≤6 months	69	77.5		63	70.0		132	73.7	0.160
>6 months	20	22.5		27	30.0		47	26.3	

*Early introduction of new foods*									
<4 months	54	60.6		36	40.0		90	50.3	<0.001
≥4 months	35	39.4		54	60.0		89	49.7	

*Did you receive advice on breastfeeding during prenatal care?*									
No, never	14	14.6		17	17.2		31	15.9	<0.01
Yes, rarely	19	19.8		38	38.4		57	29.2	
Yes, in all consultations	63	65.6		44	44.4		107	54.9	

*Professional that provided advice*									
Physician	38	46.3		77	93.9		115	70.1	<0.001
Nurse	35	42.7		3	3.7		38	23.2	
Others	9	11.0		2	2.4		11	6.71	

*After delivery, did you receive help in the breastfeeding process?*									
No	51	49.5		21	21.2		72	35.6	<0.001
Yes	52	50.5		78	78.8		130	64.4	

*Did you receive breastfeeding advice during childcare?*									
No, never	24	23.3		11	11.1		35	17.3	0.040
Yes, rarely	12	11.7		9	9.1		21	10.4	
Yes, in all consultations	67	65.0		79	79.8		146	72.3	

*Did you receive advice on how to introduce new foods to the child?*									
No, never	32	31.0		12	12.1		44	21.8	<0.001
Yes, rarely	8	7.8		1	1.0		9	4.46	
Yes, always	63	61.2		86	86.9		149	73.8	

*Professional that provided advice*									
Physician	44	62.0		84	96.6		128	81	<0.001
Nurse	21	29.6		0	0.0		21	13.3	
Others	6	8.5		3	3.4		9	5.7	

*Hours/day in front of TV*									
<2 h	18	18.0		18	18.4		36	18.2	0.042
2–5 h	33	33.0		48	49.0		81	40.9	
>5 h	49	49.0		32	32.7		81	40.9	

*Family meals in front of TV*									
Never	32	32.0		51	52.0		83	41.9	<0.001
Sometimes	17	17.0		28	28.6		45	22.7	
Always	51	51.0		19	19.4		70	35.4	

*Foods the mother gives the child that are shown on TV media*									
Never	43	46.2		47	53.4		90	49.7	<0.001
Sometimes	14	15.1		28	31.8		42	23.2	
Always	36	38.7		13	14.8		49	27.1	

a Statistical significance obtained by Chi-square test.

The children's food intake scores shown in Table 3 demonstrate that the higher the maternal educational level and household income, the higher the consumption of foods that belong to groups II (fruits and vegetables) and III (meat, viscera and eggs). The consumption of processed products was significantly higher when mothers confirmed lower educational levels and family income.

**Table 3:** Medians and interquartile ranges of food consumption scores, according to socioeconomic and maternal demographic variables, and of the children in the case group and comparison group, Maceio, AL, 2012.

Variables	Food group†																
	I			II			III			IV			V			VI	
	Med	IQ		Med	IQ		Med	IQ		Med	IQ		Med	IQ		Med	IQ
*Groups*																	
Case	3.6	1.92–4.48		2.4	0.95–4.25		1.1	0.11–1.73		2.0	1.86–2.40		1.9	0.40–3.13		2.7	0.93–5.28
Comparison	3.1	2.33–4.00		5.1	3.59–6.63		1.6	1.20–1.86		2.4	1.86–2.80		1.9	0.53–2.80		0.2	0.03–0.83
*p*-value *		0.05			<0.01			<0.01			<0.01			0.76			<0.01

*Maternal level of schooling*																	
≤4 years	3.5^a^	3.09–6.05		2.3^a^	0.50–3.85		0.4^a^	0.04–1.80		2.0	1.27–2.60		3.7	0.93–3.73		8.6^a^	2.46–9.40
5–8 years	3.7^b^	2.23–4.76		2.7^a^	1.23–4.80		1.2^b^	0.30–1.76		2.0	1.86–2.46		1.9	0.53–2.90		2.6^b^	0.70–4.53
≥9 years	2.9^c^	2.33–3.85		5.0^b^	3.67–6.57		1.5^c^	1.17–1.85		2.4	1.86–2.80		1.7	0.40–2.80		0.2^c^	0.03–0.90
*p*-value *		0.03			<0.01			0.02			0.18			0.51			<0.01

*Per capita income (MW)*																	
≤0.20	3.7	1.75–4.43		1.9^a^	0.00–3.53		0.6^a^	0.00–1.51		2.0^a^	1.86–2.40		1.9	0.53–2.81		2.6^a^	0.76–5.43
0.20–0.55	3.5	2.93–4.42		2.6^b^	1.43–4.95		1.2^b^	0.79–2.16		2.0^b^	1.86–2.40		2.0	0.13–3.73		2.7^b^	1.17–5.07
0.55–2.71	3.2	2.33–4.00		5.1^c^	3.33–6.90		1.7^b^	1.20–1.86		2.4^c^	1.86–2.80		1.9	0.93–2.90		0.7^c^	0.10–2.80
≥2.71	3.1	2.45–3.91		5.1^c^	3.71–6.40		1.5^c^	1.20–1.80		2.1^b^	1.86–2.53		1.9	0.93–2.80		0.1^d^	0.00–0.53
*p*-value *		0.37			<0.01			<0.01			0.03			0.91			<0.01

*Hours/day in front of TV*																	
<2 h	3.1	2.45–3.91		5.1^a^	3.71–6.40		1.5	1.20–1.80		2.1	1.86–2.53		1.9	0.93–2.80		0.1^a^	0.00–0.53
2–5 h	3.2	2.51–4.26		4.3^b^	2.41–6.13		1.3	0.83–1.86		2.0	1.86–2.51		1.9	0.93–2.91		0.5^a^	0.10–2.81
>5 h	3.3	2.21–4.13		3.2^c^	1.50–5.33		1.3	0.70–1.73		2.0	1.86–2.80		1.9	0.20–2.80		1.2^b^	0.21–3.93
*p*-value *		0.94			0.04			0.69			0.81			0.24			0.02

*Meals in front of TV*																	
Never	3.3	2.36–4.27		4.0^a^	2.00–6.03		1.7^a^	1.02–1.86		2.0	1.86–2.54		1.9	0.53–2.93		0.6^a^	0.10–3.14
Sometimes	3.3	2.22–4.19		4.2^b^	1.97–5.49		1.2^b^	0.56–1.70		2.3	1.86–2.80		1.9	0.53–3.20		0.7^b^	0.07–2.69
Always	3.2	2.21–4.13		2.7^c^	0.93–5.11		1.2^b^	0.33–1.73		2.0	1.86–2.80		1.9	0.45–2.60		1.6^c^	0.53–4.03
*p*-value *		0.89			0.04			0.04			0.75			0.71			0.01

*Foods shown on TV media*																	
Never	3.1	2.22–4.00		4.0^a^	1.62–5.73		1.3	0.66–1.85		1.9^a^	1.86–2.40		1.9	0.26–2.80		0.6^a^	0.07–2.40
Sometimes	3.8	2.53–4.26		4.6^b^	2.80–6.14		1.3	0.80–1.73		2.4^b^	1.86–2.90		2.1	0.53–3.20		0.7^b^	0.13–3.37
Always	3.4	2.53–4.26		2.7^c^	1.33–5.06		1.2	0.50–1.76		2.2^c^	1.86–2.80		1.9	0.93–3.73		2.7^c^	0.66–5.96
*p*-value *		0.16			0.03			0.55			0.01			0.24			<0.01

MW Med, Median; IQ, Interquartile Interval.

a,b,c,d Different letters, statistical differences between the categories.

* Kruskal Wallis Test. Subsequent test: Mann–Whitney *U*test.

† Group I: breads and cereals. Group II: fruits and vegetables. Group III: meat, viscera, eggs and beans. Group IV: Milk and dairy products. Group V: sugars, sweets, fats and oils. Group VI: processed foods considered to be superfluous.

The data in Table 3 show that if mothers had sedentary lifestyle, such as spending more time watching television, and if the families had the meals in front of the TV, their children showed lower consumption of foods from group II (fruits and vegetables) and consumed more foods from group VI (processed foods). Consumption of processed foods was also more common in children whose mothers had offered them foods shown in television advertisements.

## Discussion

The results point to low adherence to breastfeeding, lack of help and advice from health professionals regarding feeding practices of infants and inadequate food consumption in the first 2 years of life, more frequently observed in mothers belonging to families with lower income and lower education levels.

In recent years, there has been innumerable scientific evidence emphasizing the importance of exclusive breastfeeding in the first 6 months of life and the maintenance of breastfeeding until the child is at least 2 years old.^3^^,^ 14 ^,^ 15 Despite this evidence, the duration of breastfeeding in this study was ≤6 months in 70.0% of children, higher values than those reported in national literature.^14^^,^ 16 ^,^ 17 Also, it was verified a high frequency of early introduction (<4 months) of other foods, mainly among children from the case group (60.6%) versus the comparison group (40.0%, *p*<0.001). This statistically significant difference may have been caused by the lack of advice to mothers from the case group on how to introduce new foods to the children, considering that 38.8% did not receive such advice. These mothers were treated at the FHU, where health professionals are responsible for primary care and must prioritize the performance of a number of actions, at the individual and collective levels, that include the promotion and protection of health,^18^ which does not seem to have occurred. Despite the government's investment in health policies that promote prevention strategies and nutritional advice as routine practice in health care services,^19^^,^ 20 the implementation of these policies is yet to become a common practice throughout the country, which probably would be able to change this reality.

Considering this view, the practice observed at the FHU suggests lack of knowledge or inattentiveness in putting these policies to use, as more than one-quarter of the assisted mothers reported never having received advice regarding the introduction of new foods. Therefore, it seems that the activities of the FHU multidisciplinary team are focused on the performance of specific tasks and achievement of goals, or on excess of demand, which affect the quality of the care provided to the population, as observed in other studies.^21^^,^ 22

However, it is noteworthy the fact that the mother's lack of nutritional education is a common reality in Brazil, as studies show that children up to 24 months are being weaned at an increasingly earlier age and the introduction of complementary foods is occurring at the wrong way and time.^1^^,^ 5 ^,^ 6 ^,^ 23 Data from the II Breastfeeding Prevalence Research,^17^ at the Federal District and state capitals, show that infants already consume cookies and/or snacks and, in the Northeast region, this consumption included almost half of the children aged 6 months (44.1%).

In the comparison group, whose mothers had better income and educational levels, there was a higher consumption of foods from groups II and III, which are beneficial and essential to children's health, with lower consumption of processed foods. This finding suggests that the access to information that processed products are unessential in the first 2 years of life still depends on better socio-cultural level, i.e., that the nutrition of a child depends not only on the access to adequate food, but above all, on the family's educational level and culture.^24^^,^ 25

In the present study, which evaluated food consumption through scores, it was observed an agreement with other reports in the literature that evaluated the food intake of children using different methodologies. Aquino and Philippi,^23^ studying children's consumption of processed foods, assessed by counting the consumed portions, found a high sugar consumption in children whose families had lower income (1st quartile of *per capita* income R$59.19). Toloni et al.^6^ evaluated infants’ consumption of processed products through consumed portions and found that approximately two thirds of the children received foods with obesogenic potential before 12 months of life, such as instant noodles, snacks, sandwich cookies, artificial juice, soda and candy/lollipops/chocolate. The mothers who offered these foods to their infants had, at a greater proportion, lower educational level, lower income and were younger.

Another variable observed in the study and that can have a negative influence on food quality of the infants was the mother's sedentary life habits, such as spending more time watching television. In the case group, most mothers reported this behavior, which possibly contributed to the higher consumption score of processed foods among infants from this group. Studies have observed poor eating patterns in families who watch television at mealtime, with higher consumption of high-calorie foods by children, adolescents and adults. In Brazil, in Florianopolis, Fiates et al.^26^ observed that, while the family watched television, there was a higher consumption of foods that were sources of simple carbohydrates and fats, suggesting that families who watch television at mealtime can get distracted and lose control over food consumption, by ignoring internal satiety signals, which can lead to excessive food intake.^27^^-^ 29 These changes in food consumption can have a great impact on children, as good eating habits are essential for the prevention of chronic diseases and consolidation of healthy behaviors in childhood, which will extend into adulthood.^11^^,^ 30

It is noteworthy that this is a cross-sectional study in which information was provided by mothers/guardians through food recall, which can result in imprecisions about the period of introduction of new foods and duration of breastfeeding.^1^ However, this memory bias must not have substantially influenced the results, as only mothers of infants participated in this research and the introduction of new foods in the diet occurred a few months before the interview; another benefit was that data collection was carried out by two trained nutritionists. It is also worth mentioning that the data shown here do not reflect the reality of all private practices and all FHUs in the capital of Alagoas, because the study was not a population-based one. However, the study design allows us to speculate that the findings can be applied in similar contexts.

In conclusion, the results suggest that maternal socioeconomic status influences the quality of food offered to the infant, since in the case group children aged up to 24 months had already been introduced to processed foods, at the expense of healthy food consumption. The data also suggest that the influence of television media and the lack of support from health care professionals contribute to the early introduction of inappropriate food practices.

It is necessary to implement intervention measures, both in health care services and private practices, aiming to offer advice to mothers and promote healthy complementary feeding. It is necessary to assess dietary intakes in researches aimed to establish health status, as they allow characterizing the risk level and vulnerability of the population to overeating, as well adapting and proposing intervention measures to ensure health, particularly in the population group aged up to 2 years, which is the time when diet is one of the determinants of growth rate and development.
