# Supplementary material for: Socioeconomic, cultural and demographic maternal factors associated with dietary patterns of infants
Source: Rev Paul Pediatr. 2015 Oct-Dec;33(4):445–52. doi: 10.1016/j.rpped.2015.03.006 (PMC4685565; doi:10.1016/j.rpped.2015.03.006)
Supplement: Translation

# Translation: Fatores socioeconômicos, culturais e demográficos maternos associados ao padrão alimentar de lactentes

**Authors:** Andréa Marques Sotero, Poliana Coelho Cabral, Giselia Alves Pontes da Silva

**Affiliations:** a Universidade de Pernambuco (UPE), Petrolina, PE, Brasil; b Departamento de Nutrição, Universidade Federal de Pernambuco (UFPE), Recife, PE, Brasil; c Pediatria, Universidade Federal de Pernambuco (UFPE), Recife, PE, Brasil

**Keywords:** Hábitos alimentares, Consumo de alimentos, Lactente

## Abstract

**Objetivo:**

Analisar o padrão de consumo alimentar de lactentes e sua associação com variáveis socioeconômicas, culturais e demográficas maternas.

**Métodos:**

Foi feito um estudo de corte transversal que envolveu dois grupos de mães de crianças até 24 meses (n=202) residentes na cidade de Maceió-Alagoas. O grupo caso foi constituído por mães cadastradas em uma unidade de saúde da família. O grupo comparação foi constituído de mães que levaram seus filhos para atendimento em dois consultórios particulares de pediatria da cidade. O consumo alimentar foi avaliado com o uso de um questionário de frequência alimentar (QFA) qualitativo e validado. O QFA foi avaliado pelo método no qual o cômputo geral da frequência do consumo é convertido em escore.

**Resultados:**

As crianças com maior renda familiar e mães com melhor nível de escolaridade (grupo comparação) apresentaram as maiores medianas de escores de consumo dos grupos alimentares de frutas, legumes e verduras (*p*<0,01) e carnes, miúdos e ovos (*p*<0,01), quando comparadas com as crianças do grupo caso. Por outro lado, as medianas de escores de consumo de produtos industrializados foram mais elevadas entre as crianças do grupo caso (*p*<0,01).

**Conclusões:**

O nível socioeconômico materno influenciou na qualidade da alimentação que foi oferecida ao lactente, pois, no grupo caso, crianças de até 24 meses já tinham no seu cardápio produtos industrializados, em detrimento do consumo de alimentos saudáveis.

## Introdução

A alimentação adequada nos dois primeiros anos de vida é essencial, pois esse é um período caracterizado por rápido crescimento, desenvolvimento e formação dos hábitos alimentares que podem permanecer ao longo da vida.^1^^,^ 2

O aleitamento materno exclusivo até os seis meses e, após essa idade, o aleitamento materno até os dois anos ou mais, aliado à introdução oportuna da alimentação complementar (AC) balanceada e equilibrada, são enfatizados pela Organização Mundial da Saúde como medidas importantes de saúde pública, com impacto efetivo na redução do risco para o desenvolvimento de doenças crônicas não transmissíveis (DCNT), tais como: obesidade, hipertensão e diabetes melito.^1^^,^ 3 Apesar disso, estudos feitos nos últimos 30 anos mostram que a frequência das DCNT tem aumentado em todas as regiões brasileiras e classes sociais, com destaque para a obesidade infantil, com prevalências que variam entre 5 e 18%.^4^

As práticas alimentares, além de ser determinantes das condições de saúde na infância, estão fortemente condicionadas ao poder aquisitivo das famílias, pois influenciam diretamente na disponibilidade, quantidade e qualidade dos alimentos consumidos.^5^ Nos últimos anos, ocorreram mudanças nos hábitos alimentares da população, principalmente em relação à substituição de alimentos caseiros e naturais por alimentos industrializados, considerados supérfluos, com elevada densidade energética e baixa qualidade nutricional.^5^^,^ 6 Contribuíram também para essas mudanças o mercado publicitário, a globalização, o ritmo acelerado de vida nas grandes cidades e o trabalho da mulher fora do lar.^7^

A identificação dos padrões alimentares de lactentes constitui importante objeto de estudo da epidemiologia nutricional, no sentido de compreender um dos fatores responsáveis pela saúde na infância.^8^ Por outro lado, há necessidade de aperfeiçoamento da forma de avaliação do padrão alimentar por meio do uso de novas metodologias.^9^

Assim, o uso do método de frequência de consumo de alimentos, analisada por meio de escores, pode ser um instrumento útil na avaliação da qualidade alimentar oferecida ao lactente. Logo, este estudo visa a analisar o padrão de consumo alimentar de lactentes e sua associação com variáveis socioeconômicas, culturais e demográficas maternas.

## Método

Foi feito um estudo de corte transversal, que envolveu dois grupos de mães de crianças até 24 meses residentes na cidade de Maceió-Alagoas. O grupo caso foi constituído por mães cadastradas na Unidade de Saúde da Família Carla Nogueira (USF), em um bairro de baixa renda, pertencente ao VI Distrito Sanitário. O grupo comparação foi constituído de mães que levaram seus filhos para atendimento em dois consultórios particulares de pediatria da cidade.

Os critérios de inclusão foram: mães com filhos até 24 meses e que tinham aparelho de televisão em casa, pois a televisão tem se tornado o fator cultural que mais veicula mensagens sobre alimentos não saudáveis.^1^ E os critérios de exclusão foram: mães adolescentes e, ainda, para o grupo comparação, mães que tiveram a consulta paga por outro membro que não fosse da família.

A amostra foi de conveniência, composta por 202 mães de lactentes, todas atendidas nos locais escolhidos para o estudo e que, além de preencher os critérios de inclusão, assinaram o termo de consentimento livre e esclarecido, de fevereiro a abril de 2012. Das 220 mães recrutadas 202 participaram do estudo, com uma perda amostral de 8,1%. O estudo foi submetido à apreciação do Comitê de Ética em Pesquisa em Seres Humanos da Universidade Federal de Pernambuco e aprovado em 23 de janeiro de 2012, sob o número do protocolo 0437.0.172.000-11.

O tamanho da amostra foi estimado pelo *software* EPI Info, versão 3.5.1. No cálculo foi considerado que 49% das mães de baixo nível socioeconômico introduzem alimentos supérfluos precocemente, baseado no estudo de Spinelli, Souza e Souza,^10^ e 28% das mães, por terem melhor poder aquisitivo, não introduzem. Assumiu-se um risco relativo de 2,5 de não introduzir precocemente alimentos supérfluos para um nível de significância de 95% (1-α) e um poder igual a 80% (1-β). A razão entre grupo caso/comparação foi de 1:1. O valor estimado da amostra foi de 92 binômios mães-crianças lactentes em cada grupo. Foram adicionados 10% por grupo para compensar eventuais perdas, o que resultou numa amostra de 100 binômios em cada grupo. A amostra final foi de 200 mães-crianças lactentes.

A coleta de dados foi feita no primeiro semestre de 2012 por duas nutricionistas treinadas, as quais entrevistaram as mães biológicas das crianças na USF e nos consultórios. Foram coletados dados: socioeconômicos (renda familiar per capita em salário mínimo, na época R$ 622, posteriormente distribuídos em quartis), culturais (comportamento frente à televisão), demográficos (idade materna, ocupação materna, número de filhos nascidos vivos, convívio da mãe com o companheiro, chefe da família, escolaridade materna e do chefe) e de saúde da criança (aleitamento materno, alimentação complementar e apoio no pré-natal). Para este estudo, a variável "aleitamento materno" foi estabelecida com base na definição adotada pela OMS,^11^ quando a criança recebe leite materno direto da mama ou ordenhado, independentemente de receber ou não outros alimentos.

Com vistas a uniformizar o preenchimento dos formulários, elaborou-se um manual com orientações aos entrevistadores e codificação das variáveis. Os instrumentos foram previamente testados com mães que compareceram na USF 30 dias antes de se iniciarem as coletas de dados.

O consumo alimentar, variável desfecho, foi avaliado com um questionário de frequência alimentar (QFA) qualitativo validado por Colluci et al.,^12^ o qual foi adaptado para a faixa de crianças até 24 meses com a finalidade de se adequar melhor aos objetivos propostos. A avaliação do QFA se deu pelo método proposto por Fornés et al.,^13^ na qual o cômputo geral da frequência do consumo é convertido em escores.

Os escores foram estabelecidos da seguinte maneira:

1. Os alimentos foram registrados por meio do QFA composto por 63 itens alimentares e classificados em quatro categorias de frequência de consumo: raro/nunca, consumo diário, semanal ou mensal;
2. Para que a frequência de consumo de cada alimento pudesse ser adotada como frequência de consumo mensal, foi atribuído um peso a cada categoria de frequência de consumo.
3. Foi definido como valor de peso máximo (peso=1) a frequência diária de consumo. Os demais pesos foram obtidos de acordo com a seguinte equação: Peso=(1/30)×(a), em que *a* correspondeu ao número de dias consumido no mês. Quando *a* se referiu ao consumo semanal, converteu-se o número à frequência de consumo mensal, multiplicando-o por 4, considerando que o mês tem quatro semanas. Por exemplo, para um alimento consumido 4 vezes na semana, sua frequência de consumo mensal foi de 16 vezes no mês. Assim, o peso para frequência ficou: Peso=(1/30)×(16)=0,533.
4. Após o cálculo do peso da frequência de consumo de cada item, os alimentos analisados foram inseridos em seis grupos estabelecidos pelo Guia Alimentar Brasileiro para menores de 2 anos: 13 o grupo I foi composto pelos alimentos-fonte de carboidratos (pães e cereais) que compõem a base da pirâmide; o grupo II foi composto por alimentos-fontes em vitaminas, mineras e fibras (frutas, legumes e verduras); no grupo III foram inseridos os alimentos-fonte de proteína de origem animal e leguminosa (carnes, miúdos, ovos e feijão); no grupo IV foram colocados os alimentos fonte de cálcio (leite e produtos lácteos); no grupo V foram inseridos os alimentos do topo da pirâmide (açúcares, doces, óleos e gorduras) e no grupo VI os alimentos industrializados considerados supérfluos, os quais, segundo o Guia Alimentar Brasileiro para menores de 2 anos, não devem ser oferecidos as crianças dessa idade, pois em suas composições apresentam quantidades excessivas de lipídeos e/ou açúcares ou contêm substâncias indesejáveis para o consumo dessa faixa etária, como corantes e conservantes químicos (caldo de carne, sopinha industrializada, macarrão instantâneo, suco artificial, refrigerante, doce industrializado, gelatina, biscoito recheado, salgadinho, pipoca amanteigada, achocolatado, balas, cereais matinais com açúcar, chocolates, papas doce ou salgada industrializadas, linguiça, salsicha, hambúrguer e salame).

Os escores de frequência de consumo foram calculados mediante o somatório dos valores da frequência de consumo para os alimentos correspondentes a cada grupo. O escore I foi representado pelo somatório dos valores de consumo dos alimentos que compuseram o Grupo I, o escore II pelo somatório dos valores de consumo de alimentos que compuseram o Grupo II e assim sucessivamente. No entanto, tendo em vista que os grupos alimentares constituídos para esse estudo foram compostos por diferentes números de alimentos, foi considerado o escore médio de cada grupo para a caracterização do padrão de consumo alimentar.

O banco de dados foi digitado no Programa Epi Info versão 6.04 (CDC/WHO, Atlanta, GE, USA), com dupla entrada, e posterior uso do módulo *validate*para checar eventuais erros de digitação. Para as análises estatísticas, foi empregado o SPSS versão 12.0 (SPSS Inc., Chicago, IL, USA). Para comparação das frequências entre os grupos caso e comparação, foi usado o teste do qui-quadrado. Os escores de frequência de consumo alimentar, em virtude de se tratar de variáveis em escala ordinal, foram descritos sob a forma de mediana e Intervalo Interquartílico (IQ). A associação entre o consumo alimentar e as variáveis explicativas foi avaliada pelos testes U de Mann-Whitney (duas medianas) e Kruskal Wallis (mais de duas medianas) e empregou-se o teste U de Mann-Whitney a *posteriori*. Na validação das associações investigadas foi adotado o valor de *p*<0,05.

## Resultados

Foram entrevistadas 202 mães, 51% (n=103) pertencentes ao Grupo Caso, que tiveram seus filhos atendidos na Unidade de Saúde da Família (USF) Carla Nogueira, e 49% (n=99) constituintes do Grupo Comparação, que tiveram seus filhos atendidos em dois consultórios da rede particular. Na Tabela 1, encontram-se as características socioeconômicas e demográficas dos grupos do estudo.

**Tabela 1:** Variáveis socioeconômicas e demográficas, segundo o grupo caso e comparação, Maceió‐Al, 2012

Variáveis	Caso			Comparação			Total		*p* a
	n	%		n	%		n	%	
*Idade materna (anos)*									
≤28	81	78,6		26	26,3		107	53,0	<0,001
>28	22	21,4		73	73,7		95	47,0	

*Ocupação materna*									
Não trabalha/do lar	48	46,6		10	10,3		58	28,7	<0,001
Desempregado	38	36,9		15	14,4		53	26,2	
Empregado	17	16,5		74	75,3		91	45,0	

*Número de filhos nascidos vivos*									
1	32	31,1		58	58,6		90	44,6	<0,001
2 e 3	53	51,5		40	40,4		93	46,0	
>3	18	17,5		1	1,0		19	9,4	

*Mãe vive com o companheiro*									
Sim	86	83,5		90	90,9		176	87,1	0,08
Não	17	16,5		9	9,1		26	12,9	

*Chefe da família*									
Mãe	10	9,8		11	11,1		21	10,4	0,77
Pai	85	82,4		80	80,8		165	81,7	
Avó	5	4,9		3	3,0		8	4,0	
Outro	3	2,9		5	5,1		8	4,0	

*Escolaridade materna (anos)*									
<4	5	4,9		0	0,0		5	2,5	<0,001
4 a 8	96	93,2		16	16,2		112	55,4	
>8	2	1,9		83	83,8		85	42,1	

*Escolaridade do chefe (anos)*									
<4	17	16,5		0	0,0		17	8,4	<0,001
4 a 8	83	80,6		23	23,2		106	52,5	
>8	3	2,9		76	76,8		79	39,1	
*Renda familiar per capita (quartis de salários mínimos)*									
≤0,20	58	56,3		0	0,0		58	28,7	<0,001
0,20 ‐ 0,55	42	40,8		3	3,0		45	22,3	
0,55 ‐ 2,71	3	2,9		47	47,5		50	24,8	
≥2,71	0	0,0		49	50,5		49	24,3	

*Cuidador da criança*									
Mãe	99	96,1		62	62,6		161	79,7	<0,001
Empregada/Babá	0	0,0		27	27,3		27	13,4	
Outros	4	3,9		10	10,1		14	6,9	

a Significância estatística obtida pelo teste do qui‐quadrado

As mães do Grupo Caso apresentaram os menores níveis de escolaridade e renda familiar, tornaram-se mães mais jovens, bem como foi mais frequente terem mais de um filho, quando comparadas com as mães do Grupo Comparação. Essas diferenças foram significativas (*p*<0,001).

Na Tabela 2, observa-se não haver diferença entre os grupos quanto à duração do aleitamento materno, com cerca de 70% das crianças amamentadas por ≤6 meses. Por outro lado, apesar de as mães do Grupo Comparação terem recebido menos orientação sobre o aleitamento materno no pré-natal (44,4%×63%; *p*<0,01), após o parto essas mães foram as que tiveram maior ajuda no processo de amamentação (*p*<0,001) e maior volume de orientação sobre a amamentação (*p*=0,04) e a introdução de novos alimentos (*p*<0,001), quando comparadas com as mães do Grupo Caso.

**Tabela 2:** Variáveis de comportamento e de orientação nutricional relacionadas à saúde das crianças, segundo o grupo de caso e comparação, Maceió, Al, 2012

Variáveis	Caso			Comparação			Total		*p* a
	n	%		n	%		n	%	
*Idade da criança (meses)*									
≤13	56	54,4		52	52,5		108	53,5	0,452
>13	47	45,6		47	47,5		94	46,5	

*Duração do aleitamento materno*									
≤6 meses	69	77,5		63	70,0		132	73,7	0,160
>6 meses	20	22,5		27	30,0		47	26,3	

*Introdução precoce de novos alimentos*									
<4 meses	54	60,6		36	40,0		90	50,3	<0,001
≥4 meses	35	39,4		54	60,0		89	49,7	

*No pré‐natal recebeu alguma orientação sobre aleitamento materno*									
Não, nunca	14	14,6		17	17,2		31	15,9	<0,01
Sim, raramente	19	19,8		38	38,4		57	29,2	
Sim, todas as consultas	63	65,6		44	44,4		107	54,9	

*Profissional que deu as orientações*									
Médico	38	46,3		77	93,9		115	70,1	<0,001
Enfermeiro	35	42,7		3	3,7		38	23,2	
Outros	9	11,0		2	2,4		11	6,71	
*Após o parto recebeu ajuda no processo de amamentação*									
Não	51	49,5		21	21,2		72	35,6	<0,001
Sim	52	50,5		78	78,8		130	64,4	

*Recebeu orientação de amamentação na puericultura*									
Não, nunca	24	23,3		11	11,1		35	17,3	0,040
Sim, raramente	12	11,7		9	9,1		21	10,4	
Sim, todas as consultas	67	65,0		79	79,8		146	72,3	

*Recebeu orientação de como introduzir novos alimentos a criança*									
Não, nunca	32	31,0		12	12,1		44	21,8	<0,001
Sim, raramente	8	7,8		1	1,0		9	4,46	
Sim, sempre	63	61,2		86	86,9		149	73,8	

*Profissional que deu as orientações*									
Médico	44	62,0		84	96,6		128	81	<0,001
Enfermeiro	21	29,6		0	0,0		21	13,3	
Outros	6	8,5		3	3,4		9	5,7	

*Horas/dia diante da TV*									
<2 horas	18	18,0		18	18,4		36	18,2	0,042
2 a 5 horas	33	33,0		48	49,0		81	40,9	
>5 horas	49	49,0		32	32,7		81	40,9	

*Refeições da família diante da TV*									
Nunca	32	32,0		51	52,0		83	41,9	<0,001
Às vezes	17	17,0		28	28,6		45	22,7	
Sempre	51	51,0		19	19,4		70	35,4	
*Alimentos que a mãe dá ao filho são veiculados pela mídia televisiva*									
Nunca	43	46,2		47	53,4		90	49,7	<0,001
Às vezes	14	15,1		28	31,8		42	23,2	
Sempre	36	38,7		13	14,8		49	27,1	

a Significância estatística obtida pelo teste do qui‐quadrado.

Os escores de consumo alimentar das crianças apresentado na Tabela 3 demonstram que quanto maior a escolaridade materna e a renda familiar, maior o consumo de alimentos pertencentes aos grupos alimentares II (frutas, legumes e verduras) e III (carnes, miúdos e ovos). Já o consumo de produtos industrializados foi significativamente mais elevado quando as mães confirmaram menores níveis de escolaridade e renda familiar.

**Tabela 3:** Medianas e intervalos interquartílicos dos escores de consumo alimentar, segundo variáveis socioeconômicas e demográficas maternas, das crianças do grupo caso e comparação, Maceió, AL, 2012

Variáveis	Grupo de alimentos†																
	I			II			III			IV			V			VI	
	Med	IQ		Med	IQ		Med	IQ		Med	IQ		Med	IQ		Med	IQ
*Grupos*																	
Caso	3,6	1,92‐4,48		2,4	0,95‐4,25		1,1	0,11‐1,73		2,0	1,86‐2,40		1,9	0,40‐3,13		2,7	0,93‐5,28
Comparação	3,1	2,33‐4,00		5,1	3,59‐6,63		1,6	1,20‐1,86		2,4	1,86‐2,80		1,9	0,53‐2,80		0,2	0,03‐0,83
*p*‐valor*		0,05			<0,01			<0,01			<0,01			0,76			<0,01

*Escol. Materna*																	
≤4 anos	3,5^a^	3,09‐6,05		2,3^a^	0,50‐3,85		0,4^a^	0,04‐1,80		2,0	1,27‐2,60		3,7	0,93‐3,73		8,6^a^	2,46‐9,40
5 a 8 anos	3,7^b^	2,23‐4,76		2,7^a^	1,23‐4,80		1,2^b^	0,30‐1,76		2,0	1,86‐2,46		1,9	0,53‐2,90		2,6^b^	0,70‐4,53
≥9 anos	2,9^c^	2,33‐3,85		5,0^b^	3,67‐6,57		1,5^c^	1,17‐1,85		2,4	1,86‐2,80		1,7	0,40‐2,80		0,2^c^	0,03‐0,90
*p*‐valor*		0,03			<0,01			0,02			0,18			0,51			<0,01

*RPC (SM)*																	
≤0,20	3,7	1,75‐4,43		1,9^a^	0,00‐3,53		0,6^a^	0,00‐1,51		2,0^a^	1,86‐2,40		1,9	0,53‐2,81		2,6^a^	0,76‐5,43
0,20 ‐ 0,55	3,5	2,93‐4,42		2,6^b^	1,43‐4,95		1,2^b^	0,79‐2,16		2,0^b^	1,86‐2,40		2,0	0,13‐3,73		2,7^b^	1,17‐5,07
0,55 ‐ 2,71	3,2	2,33‐4,00		5,1^c^	3,33‐6,90		1,7^b^	1,20‐1,86		2,4^c^	1,86‐2,80		1,9	0,93‐2,90		0,7^c^	0,10‐2,80
≥2,71	3,1	2,45‐3,91		5,1^c^	3,71‐6,40		1,5^c^	1,20‐1,80		2,1^b^	1,86‐2,53		1,9	0,93‐2,80		0,1^d^	0,00‐0,53
*p*‐valor*		0,37			<0,01			<0,01			0,03			0,91			<0,01

*Horas/dia diante da TV*																	
<2 horas	3,1	2,45‐3,91		5,1^a^	3,71‐6,40		1,5	1,20‐1,80		2,1	1,86‐2,53		1,9	0,93‐2,80		0,1^a^	0,00‐0,53
2 a 5 horas	3,2	2,51‐4,26		4,3^b^	2,41‐6,13		1,3	0,83‐1,86		2,0	1,86‐2,51		1,9	0,93‐2,91		0,5^a^	0,10‐2,81
>5 horas	3,3	2,21‐4,13		3,2^c^	1,50‐5,33		1,3	0,70‐1,73		2,0	1,86‐2,80		1,9	0,20‐2,80		1,2^b^	0,21‐3,93
*p*‐valor*		0,94			0,04			0,69			0,81			0,24			0,02

*Refeições na TV*																	
Nunca	3,3	2,36‐4,27		4,0^a^	2,00‐6,03		1,7^a^	1,02‐1,86		2,0	1,86‐2,54		1,9	0,53‐2,93		0,6^a^	0,10‐3,14
Às vezes	3,3	2,22‐4,19		4,2^b^	1,97‐5,49		1,2^b^	0,56‐1,70		2,3	1,86‐2,80		1,9	0,53‐3,20		0,7^b^	0,07‐2,69
Sempre	3,2	2,21‐4,13		2,7^c^	0,93‐5,11		1,2^b^	0,33‐1,73		2,0	1,86‐2,80		1,9	0,45‐2,60		1,6^c^	0,53‐4,03
*p*‐valor*		0,89			0,04			0,04			0,75			0,71			0,01

*Alimentos da mídia TV*																	
Nunca	3,1	2,22‐4,00		4,0^a^	1,62‐5,73		1,3	0,66‐1,85		1,9^a^	1,86‐2,40		1,9	0,26‐2,80		0,6^a^	0,07‐2,40
Às vezes	3,8	2,53‐4,26		4,6^b^	2,80‐6,14		1,3	0,80‐1,73		2,4^b^	1,86‐2,90		2,1	0,53‐3,20		0,7^b^	0,13‐3,37
Sempre	3,4	2,53‐4,26		2,7^c^	1,33‐5,06		1,2	0,50‐1,76		2,2^c^	1,86‐2,80		1,9	0,93‐3,73		2,7^c^	0,66‐5,96
*p*‐valor*		0,16			0,03			0,55			0,01			0,24			<0,01

Escol. Materna, escolaridade materna; RPC (SM), renda per capita (salário mínimo); Refeições na TV, refeições da família diante da TV, Alimentos da mídia TV, Alimentos que a mãe dá ao filho são veiculados pela mídia televisiva; Med, Mediana; IQ, Intervalo Interquartílico.

a,b,c,d Letras diferentes, diferenças estatísticas entre as categorias

* Teste de Kruskal Wallis. Teste a *posteriori:* U de Mann‐Whitney.

† Grupo I: pães e cereais. Grupo II: frutas, legumes e verduras. Grupo III: carnes, miúdos, ovos e feijão. Grupo IV: leite e produtos lácteos. Grupo V: açúcares, doces, óleos e gorduras. Grupo VI: alimentos industrializados considerados supérfluos.

Os dados na Tabela 3 mostram que se as mães tinham hábitos de vida sedentários, como passar mais horas em frente à televisão, e se as famílias faziam refeições diante da TV, seus filhos tinham menor consumo nos grupos alimentares II (frutas, legumes e verduras) e consumiam mais alimentos do grupo VI (produtos industrializados). O consumo de produtos industrializados também foi mais frequente em crianças cujas mães ofereciam aos filhos alimentos veiculado pelas propagandas televisivas.

## Discussão

Os resultados apontam para uma baixa adesão à prática do aleitamento materno, a falta de ajuda e orientação dos profissionais da saúde no que diz respeito às práticas alimentares dos lactentes e um consumo de alimentos inadequados nos dois primeiros anos de vida, mais frequentes nas mães pertencentes às famílias de mais baixa renda e menor nível de escolaridade.

Nos últimos anos, inúmeras são as evidências científicas que enfatizam a importância da amamentação exclusiva nos primeiros seis meses de vida e a manutenção do aleitamento materno até pelo menos dois anos.^3^^,^ 14 ^,^ 15 Apesar dessas evidências, a duração do aleitamento materno neste estudo foi ≤6 meses em 70% das crianças, valores superiores aos relatados na literatura nacional.^14^^,^ 16 ^,^ 17 Foi verificada, também, elevada frequência de introdução precoce (<4 meses) de alimentos, principalmente entre as crianças do Grupo Caso (60,6%) *versus* as do Grupo Comparação (40%, *p*<0,001). Essa diferença estatisticamente significativa pode ter sido ocasionada pela falta de orientação às mães do Grupo Caso sobre como introduzir novos alimentos para as crianças. Teve-se em vista que 38,8% não receberam tal orientação. Tais mães foram atendidas na USF, onde os profissionais de saúde são responsáveis pela atenção básica e devem priorizar um conjunto de ações, nos âmbitos individual e coletivo, que abranja a promoção e a proteção da saúde,^18^^,^ 19 o que parece não ter ocorrido. Apesar do investimento do governo em políticas de saúde que promovem ações de prevenção e orientação nutricional como atividade de rotina nos serviços de saúde,^19^^,^ 20 a implantação dessas políticas ainda não é uma prática comum em todo o território nacional, o que provavelmente seria capaz de mudar essa realidade.

Sob essa ótica, a prática observada na USF em questão sugere desconhecimento ou desatenção na operacionalização dessas políticas, uma vez que mais de ¼ das mães atendidas relataram nunca ter sido orientadas no tocante à introdução de novos alimentos. Logo, parece que as atividades da equipe multiprofissional da USF estão voltadas para o desempenho de tarefas circunstanciais e cumprimento de metas, ou para o excesso de demanda, o que prejudica a qualidade do atendimento prestado à população, como observado em outros estudos.^21^^,^ 22

Contudo, chama-se atenção para o fato de que a falta de educação nutricional das mães é uma realidade comum no Brasil, uma vez que estudos mostram que as crianças até 24 meses estão sendo desmamadas cada vez mais cedo e a introdução de alimentos complementares está ocorrendo de forma errada e precoce.^1^^,^ 5 ^,^ 6 ^,^ 23 Dados da II Pesquisa de Prevalência em Aleitamento Materno,^17^ no Distrito Federal e nas capitais, mostram que lactentes já consomem bolachas e/ou salgadinhos e, no Nordeste, esse consumo atingiu quase a metade das crianças de seis meses (44,1%).

No Grupo Comparação, cujas mães tinham melhores condições de renda e níveis de escolaridade, houve o maior consumo pelos lactentes de alimentos pertencentes aos grupos II e III, que são benéficos e essenciais à saúde infantil, e houve menor consumo dos produtos industrializados. Esse achado sugere que o acesso à informação de que os produtos industrializados são dispensáveis nos primeiros dois anos de vida ainda depende do melhor nível sociocultural, ou seja, que a nutrição de uma criança não depende somente do acesso a uma alimentação adequada, mas, sobretudo, da educação e cultura da família.^24^^,^ 25

No presente estudo, no qual se avaliou o consumo por meio de escores, observou-se concordância com outros relatos na literatura que avaliaram o consumo alimentar de crianças com diferentes metodologias. Aquino & Philippi,^23^ que estudaram o consumo infantil de alimentos industrializados avaliados pela contagem de porções consumidas, encontraram um alto consumo de açúcar em crianças cujas famílias tinham menor poder aquisitivo (1° quartil de renda *per capita* R$ 59,19). Toloni et al.^6^ avaliaram o consumo por lactentes de produtos industrializados por meio da porção consumida e encontraram que aproximadamente ⅔ das crianças receberam antes dos 12 meses alimentos com potencial obesogênico, como macarrão instantâneo, salgadinhos, bolachas recheadas, suco artificial, refrigerante e bala/pirulito/chocolate. As mães que ofereceram esses alimentos tinham, em maior proporção, baixa escolaridade, menor renda e eram mais jovens.

Outro fator observado no estudo e que pode ter uma influência negativa na qualidade alimentar dos lactentes foi o hábito de vida sedentário das mães, como passar mais tempo diante da televisão. No Grupo Caso, a maioria das mães relatou esse comportamento, o que, possivelmente, contribuiu para um escore de consumo maior de produtos industrializados entre os lactentes desse grupo. Estudos têm observado, nas famílias que veem televisão no horário da refeição, padrões alimentares inadequados, com maior consumo de alimentos calóricos em crianças, adolescentes e adultos. No Brasil, em Florianópolis (SC), Fiates et al.^26^ notaram que enquanto a família assistia à televisão ocorreu maior consumo de alimentos fonte de carboidratos simples e gorduras. Isso sugere que as famílias que assistem à televisão enquanto fazem as refeições podem ficar distraídas e perder o controle em relação ao consumo de alimentos por ignorar os sinais internos de saciedade, o que pode levar ao consumo alimentar excessivo.^27^^-^ 29 Tais alterações no consumo alimentar podem ter grande impacto entre crianças, pois os bons hábitos alimentares são essenciais para a prevenção de doenças crônicas e a instalação de comportamentos saudáveis na infância, que se estendem até a vida adulta.^11^^,^ 30

Vale ressaltar que este é um estudo de corte transversal em que as informações foram dadas pelas mães/responsáveis de forma recordatória, o que pode gerar imprecisões sobre o período de introdução dos novos alimentos e a duração do aleitamento materno.^1^ No entanto, tal viés de memória não deve ter influenciado substancialmente os resultados, uma vez que só participaram desta pesquisa mães de lactentes e a introdução de novos alimentos na dieta ocorreu alguns meses antes da entrevista; outro benefício foi que a coleta de dados foi feita por duas nutricionistas treinadas. Vale ainda ressaltar que os dados aqui apresentados não refletem a realidade de todos os consultórios particulares e das USF da capital de Alagoas, pois o estudo não foi de base populacional. Mas o delineamento adotado permite especular que as conclusões possam ser aplicadas em contextos semelhantes.

Em conclusão, os resultados do estudo sugerem que o nível socioeconômico materno influencia na qualidade da alimentação oferecida ao lactente, pois, no Grupo Caso, as crianças de até 24 meses já contavam, em seu cardápio, com produtos industrializados em detrimento do consumo de alimentos saudáveis. Os dados sugerem ainda que a mídia televisiva e a falta de apoio dos profissionais da saúde contribuem para a introdução precoce de práticas alimentares inadequadas.

Há necessidade da implantação de medidas de intervenção tanto nos serviços de saúde como nos consultórios particulares com o objetivo de orientar as mães e promover uma alimentação complementar saudável. Torna-se necessário avaliar o consumo alimentar em pesquisas destinadas a estabelecer condições de saúde, pois permite caracterizar o nível de risco e a vulnerabilidade da população aos excessos alimentares, assim como adequar e propor medidas de intervenção que garantam a saúde, particularmente no segmento da população até dois anos, idade na qual a dieta constitui um dos fatores determinantes da velocidade de crescimento e desenvolvimento.
